# Development of an *in vitro *microfluidic model to study the role of microenvironmental cells in oral cancer metastasis

**DOI:** 10.12688/f1000research.131810.2

**Published:** 2024-01-15

**Authors:** Alice Scemama, Sophia Lunetto, Artysha Tailor, Stefania Di Cio, Matthew Dibble, Julien Gautrot, Adrian Biddle

**Affiliations:** 1 Blizard Institute, Queen Mary University of London, London, E1 2AT, UK; 2 Queen Mary University of London, London, England, UK; 3 School of Engineering and Materials Science, Queen Mary University of London, London, E1 4NS, UK

**Keywords:** microfluidic, cancer, metastasis, vasculature, HUVEC, microenvironment, chip

## Abstract

Metastasis occurs when cancer cells leave the primary tumour and travel to a secondary site to form a new lesion. The tumour microenvironment (TME) is recognised to greatly influence this process, with for instance the vascular system enabling the dissemination of the cells into other tissues. However, understanding the exact role of these microenvironmental cells during metastasis has proven challenging. Indeed, *in vitro* models often appear too simplistic, and the study of the interactions between different cell types in a 3D space is limited. On the other hand, even though *in vivo* models incorporate the TME, observing cells in real-time to understand their exact role is difficult. Horizontal compartmentalised microfluidic models are a promising new platform for metastasis studies. These devices, composed of adjacent microchannels, can incorporate multiple cell types within a 3D space. Furthermore, the transparency and thickness of these models also enables high quality real-time imaging to be performed. This paper demonstrates how these devices can be successfully used for oral squamous cell carcinoma (OSCC) metastasis studies, focusing on the role of the vascular system in this process. Conditions for co-culture of OSCC cells and endothelial cells have been determined and staining protocols optimised. Furthermore, several imaging analysis techniques for these models are described, enabling precise segmentation of the different cell types on the images as well as accurate assessment of their phenotype. These methods can be applied to any study aiming to understand the role of microenvironmental cell types in cancer metastatic dissemination, and overcome several challenges encountered with current *in vitro* and *in vivo* models. Hence, this new *in vitro* model capable of recapitulating important aspects of the cellular complexity of human metastatic dissemination can ultimately contribute to replacing animal studies in this field.

Research highlights **Scientific benefits**

• Development of a new *in vitro* model to study OSCC metastasis via the vascular system
• Co-culture of different cell types in a 3D matrix, making it more biologically relevant than existing models
• Ability to produce an ‘all human’ cellular environment – more disease relevant
• High quality imaging, including the ability to study metastatic events in real time at the cellular level

**3Rs benefits**

• Replacement of animal models for metastasis studies, which are moderate to severe procedures (including chemically induced and xenograft mouse tumour models)

**Practical benefits**

• Use of cell lines, allowing better reproducibility
• Number of channels and overall design of the device can be amended by the user

**Current applications**

• Disease modelling of OSCC metastasis
  ○ Investigating interaction of endothelial and OSCC cells in metastasis
  ○ Modelling progression of metastasis

**Potential applications**

• Scale-up the complexity by adding more cells of the tumour microenvironment (e.g. immune cells, lymphatic cells, etc.) as well as additional ECM types, to understand the role of the tumour microenvironment in controlling tumour metastatic behaviour
• Use patient samples to allow a more personalised approach to cancer treatment development
• Use for testing the efficacy of novel drug compounds and/or combinations

## Introduction

Head and neck squamous cell carcinoma (HNSCC) is the sixth most common malignancy worldwide ( Johnson *et al.*, 2020), of which the major sub-type oral squamous cell carcinoma (OSCC) accounts for more than 300,000 cases every year ( Choi and Myers, 2008). HPV infections remain a significant driver of OSCC ( Jiang and Dong, 2017), as well as tobacco smoking and alcohol misuse. These induce genetic changes in the cells of the oral cavity, eventually leading to the formation of a carcinoma ( Rivera, 2015; Scully and Robinson, 2016). OSCC is a type of cancer recognised to have a high impact on the patient’s quality of life, as aesthetic defects, as well as chewing and swallowing disabilities, can be induced by the disease and subsequent treatments administered ( Rivera, 2015; Sasahira and Kirita, 2018). No radical change has been observed in the treatment landscape for OSCC over the past few decades, with surgery, radiotherapy and chemotherapy remaining the main options offered to patients ( Kerawala *et al.*, 2016). Hence, the overall five-year survival for OSCC has remained at around 50% ( Scully and Robinson, 2016; Sasahira and Kirita, 2018).

More than 50% of patients with OSCC experience metastasis ( Choi and Myers, 2008), with the primary site being the lymph nodes of the neck ( Pisani *et al.*, 2020). Metastasis is a complex process where cancer cells must detach from the primary tumour and reach the lymphatic system and/or the blood system to travel to the lymph node and/or distant sites, such as the lungs, bones and liver ( Pisani *et al.*, 2020). Hence, understanding how these cancer cells reach and utilise the vascular system to create these deadly secondary tumours would allow the development of better targeted treatments which prevent this state of the disease from being reached.

*In vivo* models have been extensively used for OSCC studies. For instance, exposing rodents to molecules including 4-Nitroquinolone oxide (4NQO) or Dimethyl-1,2,benzanthracene (DMBA) can mimic the effect of tobacco and lead to the development of tumours similar to human OSCC ( Ishida *et al.*, 2017; Sequeira *et al.*, 2020). Genetically engineered mice have also been utilised with, for instance, Vitale-Cross *et al.* (2004)’s mouse model overexpressing K-ras via keratin 5 and keratin 14 promoters, leading to the development of oral lesions ( Vitale-Cross *et al.*, 2004). Xenografts have also been extensively used for metastasis studies, as these have enabled direct study of human cancer cells in immunocompromised rodents. However, the absence of the tumour’s native environment and the lack of immune cells in these models remain significant limitations of xenografts for metastasis studies. Furthermore, all these *in vivo* models have additional limitations, including their poor capture of physiological and pathological processes occurring in a human context, ethical drawbacks, high costs, length of studies and the challenges with imaging ( Scemama, Lunetto and Biddle, 2022).

Unlike *in vivo* models, *in vitro* models allow high throughput studies to be performed, at a lower cost and without ethical drawbacks. For instance, by forming an endothelial layer on a membrane, transwells have enabled assessment and quantification of cancer cell ability to cross this vascular barrier ( Katt *et al.*, 2016). However, real-time imaging of these events remains challenging with these assays, primarily due to issues with barrier opacity and the requirement to image through the construct. More complex *in vitro* models have been developed over the past few years, notably with the emergence of commercialised 3D matrices. For instance, spheroids, recognised as aggregation of cancer cells, present features of tumours and integrate cancer cell-cell interactions in a 3D environment ( Katt *et al.*, 2016; Suryaprakash *et al.*, 2020). However, despite the advances of the *in vitro* field, the study of spatial organisation and interactions between different cell types in a 3D space remains limited.

Microfluidic devices are a promising tool for metastasis studies. Since the development of the lung-on-a-chip model ( Huh *et al.*, 2010), multiple types of microfluidic devices have emerged in the biomedical research field, including microfluidic membranes, microfluidic scaffolds, microfluidic hydrogels, organ-on-a-plate and horizontal compartmentalised microfluidic devices ( Zhang *et al.*, 2018). This article focuses on horizontal compartmentalised microfluidic devices, as these appear highly suited for the study of the interactions between OSCC cells and the vasculature during metastasis. Indeed, these devices are composed of adjacent channels in which different cell types and extracellular matrices (ECMs) can be added. The level of complexity of these models can be modulated by the users, therefore enabling assessment of the effect of specific cells, matrix components and secreted proteins on the cancer cells’ phenotype simultaneously or independently ( Scemama, Lunetto and Biddle, 2022). Furthermore, the small size of these chips allows high quality imaging to be performed and interactions between cancer cells and microenvironmental cells can be followed in real-time.

Microfluidic devices remain a relatively new model in the biomedical research field, and require the introduction of materials, such as polydimethylsiloxane (PDMS), with inherently abnormal bioactivity compared to more traditional hydrogels such as collagen or fibrin matrices. Therefore, careful optimisation of the assay is required ( Rothbauer, Zirath and Ertl, 2018; Sosa-Hernández *et al.*, 2018). This paper describes how this model has been optimised for the co-culture of human endothelial cells with human OSCC cell lines in a three-channel microfluidic device, to better understand the role of the interactions between these two cell types during OSCC metastasis. This methods paper is linked with an associated research article, now available as a pre-print ( Scemama *et al.*, 2024), where we use horizontal microfluidic devices to elucidate the effect of the vasculature on the invasive behaviour of OSCC cancer stem cells.

## Methods

### HUVEC cell culture

Human umbilical vein endothelial cells (HUVECs, **Lonza**, cat. no. **C2519A; RRID**: CVCL_2959) were obtained from Lonza and cultured in EGM-2 medium (Promocell) at 37°C with 5% CO _2_. Cells were passaged when a 70–80% confluence was reached. To passage the cells, a wash with phosphate buffered saline (PBS) was first performed and cells were detached using 0.05% Trypsin-EDTA 1X (2.5 ml per T75 flask, Sigma) for 1.5 minutes. EGM-2 was then added to neutralise the trypsin. Cells were used until passage six.

### OSCC cell culture

OSCC cells (see Table 1 below for details) were cultured at 37°C with 5% CO _2_ in Dulbecco's Modified Eagle Medium (DMEM)-F12 (1:1) + GlutaMAX (31331093, Thermofisher Scientific), supplemented with 10% Fetal Bovine serum (FBS, FB-1001, Biosera), 1% Penicillin-Streptomycin (Pen-strep, Sigma-Aldrich) and 1% RM+ (composed of 10 ng/ml Epidermal Growth Factor [EGF-1, Serotec], 10 ^-10^ M Cholera Toxin [Sigma-Aldrich], 0.4 μg/ml Hydrocortisone [Sigma-Aldrich], 5 μg/ml Insulin [Sigma-Aldrich], 5 μg/ml Transferrin [Sigma-Aldrich], 2×10 ^-11^M 3,3′,5-Triiodo-L-Thyronine Sodium Salt [Sigma-Aldrich] in DMEM-F12 medium). Cells were passaged when a 70% confluence was reached, by washing them with PBS and detaching them using 0.05% Trypsin-EDTA 1X (Sigma). Once detached, growth medium was added to neutralise the trypsin. Cells were used only at low passage from frozen stocks (up to approx. passage eight), and discarded if gross changes in cell and colony morphology in tissue culture were observed. Retroviral transduction for production of a GFP tagged CA1 OSCC cell line was performed previously ( Gemenetzidis *et al.*, 2015).

**Table 1.** Cell line details.

Cell line	Description
**CA1**	Human cell line derived from OSCC by Ian Mackenzie lab *Location:* floor of the mouth *HPV:* negative Also available with GFP tag

### Microfluidic chip fabrication

**Figure 1.**
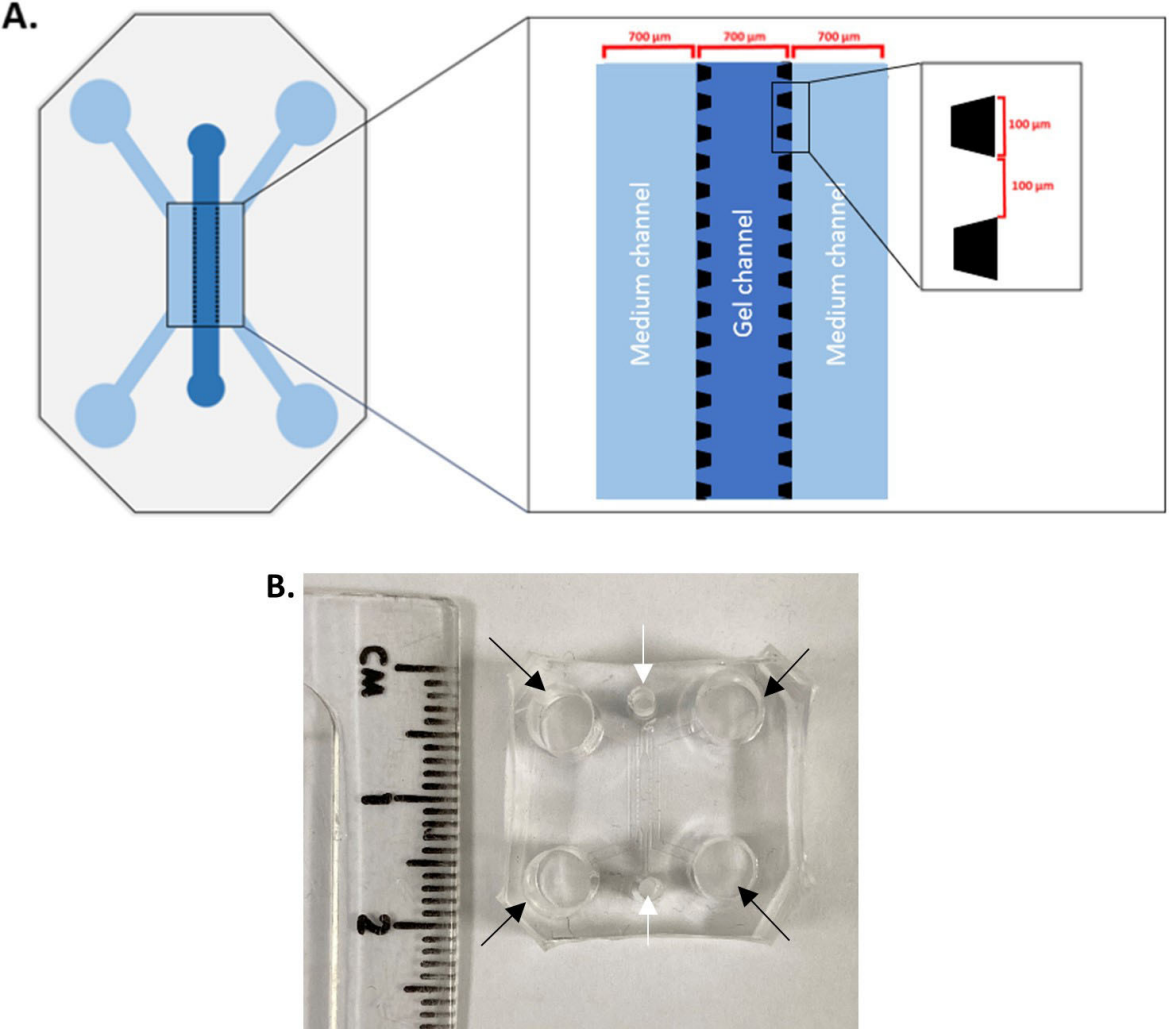
Microfluidic device structure. A. 3-channel microfluidic chip device. Medium is added in the side channels whereas the gel is added in the middle channel. Each channel width is 700 μm, and the height is 75 μm; this enables a minimal working distance from the coverslip. The side channels are delineated from the middle channels by an array of posts, which are 100 μm large and 100 μm apart. B. A cut out PDMS block with side channel inlets (black arrows) and central inlets (white arrows), showing the size of the microfluidic device. It will be bonded to a glass cover slip on the bottom.

The microfluidic chip pattern was designed on AutoCAD ^®^ and printed as a film photomask. The device was composed of three channels (700 μm width, ~75 μm height), separated from each other by an array of trapezoidal posts which prevent gel leakage into the side channels whilst allowing cells and signalling molecules to move freely across (100 μm base, 100 μm apart, Figure 1A).

To fabricate the device, a silicon wafer bearing the positive pattern of the chip was generated by photolithography. First, the wafer (PI-KEM) was washed, using acetone and isopropan-2-ol, and dried with nitrogen gas. The wafer was then spin-coated with SU-8 2050 photoresist (Kayaku Advanced Materials), by using the following spinning protocol: 500 RPM for 15 seconds with an acceleration of 100 RPM/s and 1700 RPM for 36 seconds with an acceleration of 300 RPM/s. Soft bake was performed by heating the wafer at 65°C for five minutes and 95°C for 15 minutes. The photomask, bearing the pattern of the chip, was placed on the wafer and exposed to UV light (45 mW/cm ^2^) for one second. The wafer was then heated at 65°C for five minutes and 95°C for 10 minutes, before being immersed in the developing solution, propylene glycol methyl ether acetate (PGMEA, Sigma-Aldrich), for a maximum of five minutes. Finally, a hard bake was conducted by exposing the wafer at 150°C for three minutes.

The resulting silicon wafer bearing the pattern of the desired chip was placed in a petri dish, covered with PDMS and left to set at 80°C. Once crosslinked, the PDMS block was cut out so that new PDMS could be poured onto the silicon wafer, to make new blocks. Blocks were cleaned using ispropan-2-ol and sealed to a glass coverslip via plasma bonding, to close the microchannels. Devices were placed at 80°C for three days, to allow the hydrophobicity of the PDMS to recover, and autoclaved before use for cell culture assays.

### Cell culture in the microfluidic device

**Figure 2.**
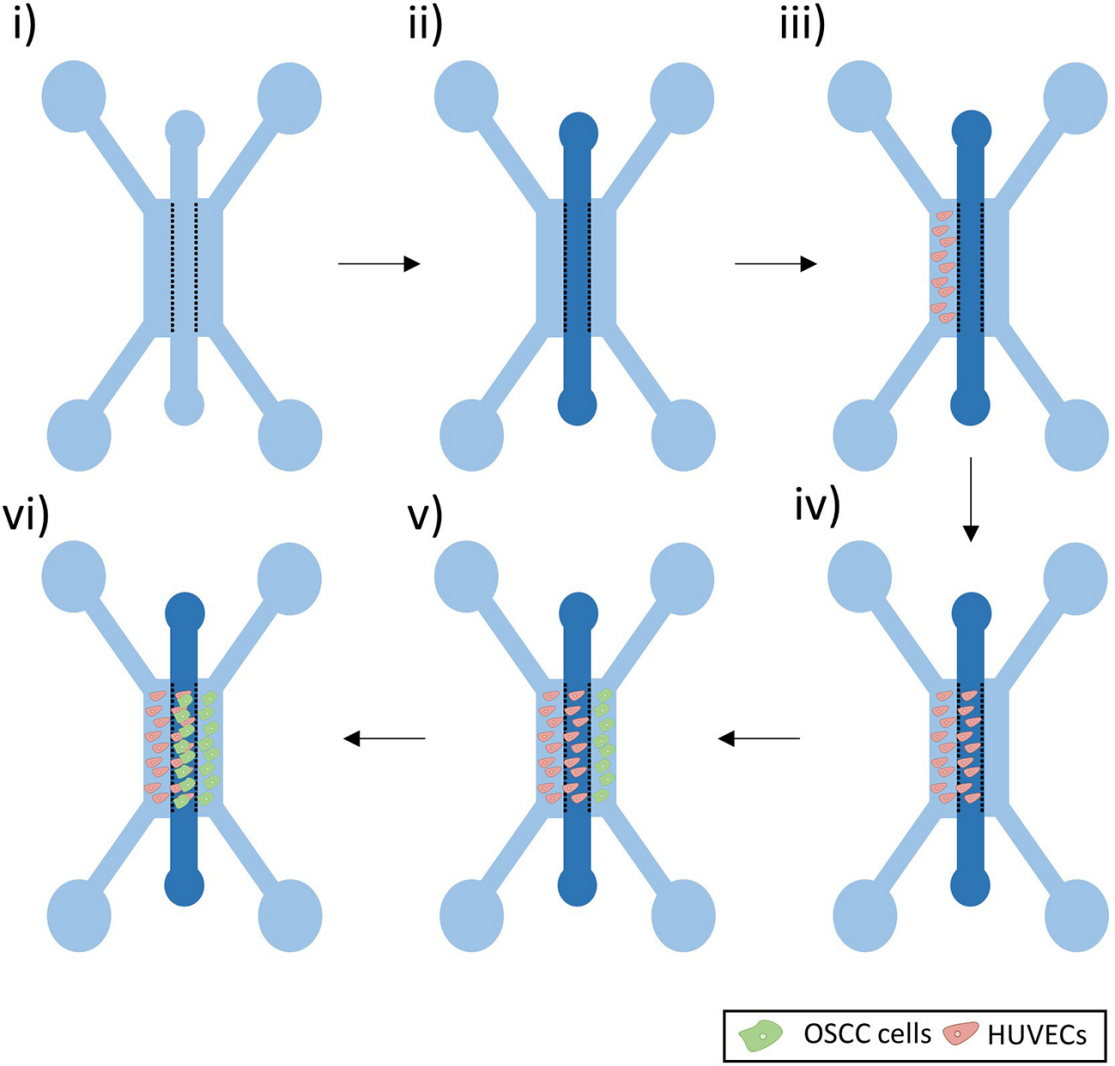
Co-culture experiment set-up. i) Microfluidic Device. ii) A fibrin gel was added in the middle channel. iii-iv) HUVECs were seeded in the left side channel and left to grow in the fibrin gel of the middle channel. v-vi) OSCC cells were then seeded in the right side channel and left to grow in the middle channel.

A fibrin gel was prepared using fibrinogen from bovine plasma (10–15 mg/ml in PBS, Sigma) and thrombin from bovine plasma (5 U/ml in PBS, Sigma-Aldrich). The gel was added in the middle channel via one of the two inlets and pipetted down at a slow pace to prevent leakage ( Figure 2i, ii). The gel was left to set at 37°C for 30 minutes, before adding medium to the side channels.

As bubbles may assemble around the posts soon after the addition of medium to the side channels, the devices were left at 37°C overnight to allow these bubbles to dissolve. Medium was then aspirated from all the side channels inlets and 8 μl of a 5 million cells/ml HUVECs suspension was added to one of the inlets of the left side channel ( Figure 2iii). To allow cells to adhere to the gel interface, the devices were flipped at 90° for 30 minutes. EGM-2 medium was supplemented with VEGF (50 ng/ml, Peprotech) and added to all the side channels. To enhance sprouting of the HUVECs, to form a developing vascular network within the gel, a flow against the cells was set up by increasing the volume of medium added in the opposite side channel (110 μl of medium in the inlets of the right side channels and 90 μl of the medium in the inlets of the HUVECs side channel). Medium was replaced every 24 hours.

When a vasculature was formed (after approx. four days) ( Figure 2iv), OSCC cells were added in the opposite side channel ( Figure 2v). Medium was aspirated from all the side channel inlets and 8 μl of a 5 million cells/ml OSCC solution was added to one of the inlets of the right side channel. To allow cells to adhere to the gel interface, the devices were flipped at 90° for 30 minutes. EGM-2 medium supplemented with VEGF (50 ng/ml, Peprotech) was added to both medium channels. Note that DMEM-F12 was not used in the co-culture, to allow the maintenance of endothelial cells in the device.

When the desired time-point of co-culture was reached ( Figure 2vi), cells were washed twice with PBS and fixed by adding 100 μl of 4% paraformaldehyde (PFA) in all the inlets for 15 minutes. Cells were washed twice with PBS and left in PBS at 4°C until stained and imaged.

### Staining and imaging

Staining reagents were added into both medium channels. Cells were permeabilised with 0.1% Triton X-100 (Sigma-Aldrich) for 10 minutes and blocked for three hours (3% BSA in PBS), at room temperature. If stained with phalloidin (1/500, Scientific Laboratory Supplies) and DAPI (1/1000 in PBS from 1 mg/ml stocks, 10236276001, Roche) or just DAPI, these stains were mixed with the blocking buffer directly and added in each microfluidic chip inlet (100 μl per inlet) for one hour, at room temperature. Cells were washed twice with PBS and left in PBS at 4°C until imaged. When conjugated antibodies were used (see Table 2, below), the antibody mix was prepared using blocking buffer. After the blocking step, 60 μl of the antibody mix was added in each inlet of the microfluidic chips and left overnight at 4°C. Cells were then stained with DAPI for one hour, washed twice with PBS and left in PBS at 4°C until imaged.

**Table 2.** Antibody details.

Antibody	Clone	Fluorophore	Company	Reference	RRID	Dilution
Vimentin	V9 (mouse monoclonal)	Alexa Fluor 488	Abcam	ab195877	AB_2916318	1:500
Pan-Cytokeratin	C-11 (mouse monoclonal)	Alexa Fluor 647	Biolegend	628604	AB_2563652	1:100

Imaging was performed using the IN Cell Analyzer 6000 (GE healthcare). Five to six fields of views, using the 20× magnification, were taken to cover the entire middle channel.

### Detailed protocols

Photolithography

Please note: SU8-2050 is light sensitive, steps 1 to 6 need to be conducted in the dark and under a fume hood

1. Clean the silicon wafer (WAFER-SILI-0006W25, PI-KEM) with acetone (100%, 8003, Avantor) and isopropan-2-ol (>99.98%, 59300M, Sigma-Aldrich) and dry it with N _2_ gas.
2. Add SU8-2050 (Y111072 0500L1GL, Kayaku Advanced Materials) in the centre of the silicon wafer and slightly tilt at different angles to spread the SU8-2050 on ~70% of the silicon wafer.
3. Place the silicon wafer in the spin coater machine (SPIN150i/200i infinite spin coater, Polos) and start the following programme:
  - Step 1: 500 revolutions per minute (RPM), 15s, acceleration: 100 RPM/s
  - Step 2: 1750 RPM, 36s, acceleration: 300 RPM/s
4. Place the silicon wafer on a heating plate (Isotemp ^®^, *fisher*brand) at 65°C for five minutes and 95°C for 15 minutes. Ensure the silicon wafer is kept in the dark as SU8-2050 is light-sensitive.
5. Place the photomask (bearing the desired chip design) on the silicon wafer and put it in the UV machine (UV-KUB 6) for one second (exposure: 100%; ~45 45 mW/cm ^2^).
6. For the post-exposure bake, place the silicon wafer on a heating plate at 65°C for five minutes and 95° for 10 minutes.
7. Under a fume hood, immerse the silicon wafer in the developing solution, PGMEA (484431, Sigma-Aldrich). If the silicon wafer is immersed in it for too long, it will become over-developed and the chip design will disappear. It is preferred to immerse the silicon wafer for 1.5 minutes and then use a squeeze bottle to better target the areas that need to be developed. During this process, regularly wash the silicon wafer with isopropanol.
8. For the hard bake, place the silicon wafer bearing the chip design on a heating plate at 150°C for three minutes.

PDMS pouring and plasma bonding

1. Place the wafer with the desired chip design in a petri dish.
2. Mix polydimethylsiloxane (PDMS; SYLGARDTM 184 Silicone Elastomer Base, Dow Chemical) with its curing agent (SYLGARDTM 184 Silicone Elastomer Curing Agent, 1:10 curing agent to PDMS) and pour it in the petri dish (~1 cm thick). Ensure this is combined well. Once satisfactorily combined, place in a desiccator for 30 minutes for degassing, at room temperature.
3. Pour this in the petri dish (~1 cm thick).
4. Place the petri dish in an oven at 80°C for at least 1.5 hours to allow the PDMS to crosslink.
5. Cut out the solidified blocks of PDMS bearing the chip pattern, using a scalpel, use punch biopsy equipment to create inlets, e.g. 5 mm punch biopsy for side channel inlets and 2 mm punch biopsy for central channel inlets (see Figure 1B).
6. Clean the PDMS blocks by first removing the dust using tape and then immersing them in dH _2_O and isopropan-2-ol sequentially and drying them with N _2_ gas in a fume hood.
7. Place the PDMS blocks and 24 × 24 mm (thickness 0.13–0.16 mm) borosilicate glass cover slips in the plasma machine (HPT-200, Henniker Plasma machine) for one minute and seal the channels by placing the PDMS and glass in full contact whilst maintaining a light pressure for 1s.
8. Incubate the devices at 80°C for 72 hours to allow the hydrophobicity to be recovered.
9. Autoclave the devices before using them for cell-based experiments.

Addition of the gel in the microfluidic chips

1. Dilute the fibrinogen type I-S from bovine plasma (F86-30, Sigma) in sterile phosphate buffered saline (PBS) to reach the desired concentration (10–20 mg/ml depending on the fibrinogen batch, each tested to determine the concentration that maintains a consistent degree of angiogenic sprouting) and leave the Eppendorf in the water bath at 37°C for one hour.
2. Filter the fibrinogen solution with a 0.22 μm filter to sterilise it.
3. Prepare the thrombin (T6634, Sigma) by diluting it in sterile PBS to reach the desired concentration (5 U/ml)
4. In a 0.5 ml Eppendorf, add 10 μl of the fibrinogen solution and then add 10 μl of the thrombin solution. Mix it by doing two up-and-downs with the pipette and slowly pipette 10 μl into one of the inlets of the middle channel. Note that less than 10 μl is needed to fill the middle channel, but using higher volumes enables the user to pipette more slowly into the inlet and therefore prevents leakage of the gel to the side channels.
5. Leave the gel to set for 30 minutes at 37°C.
6. Add 100 μl of medium to the top inlets of the side channels.
7. To ensure medium goes through the side channel, cut a 200 μl pipette tip and use it to aspirate the medium via the bottom inlet.
8. Add 100 μl of medium to the bottom inlets of the side channels. At this stage, bubbles may form alongside the gel interface. Leave the microfluidic chips overnight at 37°C to allow the bubbles to disappear before adding the cells into the devices.

Addition of the cells in the microfluidic chips

1. Transfer your cell suspension into a 50 ml falcon and centrifuge it at 1200 RPM for five minutes.
2. Re-suspend in 5 ml of growth medium and count the cells (e.g. using a haemocytometer).
3. Centrifuge the cell solution at 1200 RPM for five minutes and re-suspend in growth medium to reach a final concentration of 5 million cells/ml (note that this may vary depending on the cell type used and desired length of the assay).
4. Remove and discard the growth medium from the inlets of the side channels of the microfluidic chips using an aspirator. All the medium must be aspirated but the channels must not become dry (if dry, the cells will not move towards the gel interface and instead stay in the inlet).
5. Add 8 μl of the cell solution in one of the inlets of a side channel and rotate the device such that the long axis of the channels is parallel with the workbench and the cell solution inlet is superior, in the vertical plane, to the inlets of the opposite side channel. Ensure the device remains in this vertical position for 30 minutes at room temperature to allow the cells to adhere to the gel interface.
6. If the cells have adhered homogeneously to the gel interface, add growth medium in all the inlets of the side channels. In some instances, an interstitial flow can be added, for instance to enhance sprouting of endothelial cells. In that case, a higher volume of growth medium must be added in one of the side channels (for HUVECs: 110 μl in the inlets of the opposing side channel and 90 μl in the inlets of the cells’ side channel).
7. Place the devices at 37°C and 5% CO _2_.
8. Replace the growth medium every 24 hours.
9. Repeat steps 1–8 to place an additional cell population in the opposite side channel (e.g. cancer cells).

### Developer toolbox (GE Healthcare, version 1.10) protocols

In the following, the parameters for the two image analysis protocols in Developer Toolbox (GE Healthcare) are described, allowing the reader to recreate the protocol in the software. Applying a step-by-step process, the software allows the user to interactively adapt parameters to achieve optimal segmentation. In each step, a specific target is segmented using the user’s chosen algorithm, then postprocessing steps are applied to refine the segmentation, and lastly measures for the target are identified. Once target objects are segmented, they can be used to identify other targets in the following steps.

Segmentation algorithms include object segmentation, nuclear segmentation, and intensity segmentation. Object segmentation is kernel-based; kernel size and intensity sensitivity (i.e. minimum object brightness-to-background brightness ratio) are chosen by the user. The nuclear segmentation algorithm segments into rounded or octagonal objects using a minimum target area and sensitivity given by the user. Lastly, intensity segmentation identifies objects based on an intensity range defined by the user allowing any object size.

Post-processing steps used here include binary erosion, clump breaking, hole filling and a binary sieve. Binary erosion allows the user to smooth object boundaries using a kernel. Clump breaking utilises a second target to create segmentation between objects. For example, when clump breaking is applied to the ‘GFP cells’ target, the distance between the nuclei targets is calculated and segmentations are made at equal distances. Hole filling removes exclusions from objects, and the sieve removes objects larger or smaller than a user-defined area threshold.

After object segmentation of various targets, targets are linked to create the final cell objects. In this case, cell targets are linked to the nuclei targets. Any cell object that does not overlap with a nucleus object by at least 80% is removed, to ensure that all cell objects are nucleated.

Protocol 1

The following protocol was used in image analysis to segment two cell types in each field of view. One cell type was GFP-tagged ( *GFP cells*). All the cells were stained with phalloidin (red fluorophore). Hence, two groups of cells were segmented: the red-only cells and the red and green cells.

Here, a ‘seed’ target is used to optimise nuclei segmentation. In this case, a seed target describes a small object located at the brightest part of the nucleus. By using a seed, the algorithm receives a starting point for nuclei segmentation, which improves segmentation performance compared to seedless segmentation. Next, GFP-positive cells were segmented based on their GFP fluorophore intensity. Then, all cells were segmented ( *Cells*) based on the phalloidin intensity. GFP-negative cells were segmented by subtracting the *GFP cells* target from the *Cells* targets.

***Target sets***

• *Seed*
  • Channel: DAPI
  • Object
    ▪ Kernel size: 15
    ▪ Sensitivity: 50
  • Post processing
    ▪ Erosion (Binary):
      ▪ Kernel size: 16
    ▪ Sieve (Binary) - greater than: 20 pixels
• *Nuclei*
  • Channel: DAPI
  • Nuclear segmentation
    ▪ Minimum target area: 600 pixels
    ▪ Sensitivity: 15
  • Post processing
    ▪ Clump breaking – second segmentation: seed
    ▪ Fill holes
    ▪ Sieve (Binary) - less than
    ▪ Sieve (Binary) - greater than
  • Measure
    ▪ Sum of Nuclei
• *GFP cells*
  • Channel: FITC
  • Intensity segmentation
  • Post processing
    ▪ Erosion (Binary)
    ▪ Clump breaking – second segmentation: nuclei
    ▪ Fill holes
    ▪ Sieve (Binary) - less than
    ▪ Sieve (Binary) - greater than
• *Debris*
  • Intensity segmentation
  • Post processing
    ▪ Sieve (Binary) - less than
• *Cells*
  • Intensity segmentation
  • Post processing
    ▪ Erosion
    ▪ Clump breaking – second segmentation: nuclei
    ▪ Fill holes
    ▪ Sieve (Binary) - less than
    ▪ Sieve (Binary) - greater than
• *GFP negative cells*
  • Pre-processing macro – P.Sub_11_10_9*
  • Intensity segmentation: Minimum (1); Maximum (65535)
  • Post processing
    ▪ Sieve (Binary) - greater than
• *Whole GFP negative cells*
  • Measure
    ▪ Count Whole GFP negative cells
    ▪ Sum count Whole GFP negative cells
    ▪ Area covered by Whole GFP negative cells
    ▪ Sum area covered by Whole GFP negative cells
• *Whole GFP positive cells*
  • Measure
    ▪ Count Whole GFP positive cells
    ▪ Sum count Whole GFP positive cells
    ▪ Area covered by Whole GFP positive cells
    ▪ Sum area covered by Whole GFP positive cells

***Target linking***

• *Whole GFP negative cells*
  ▪ Primary target set: Nuclei
  ▪ Secondary target set: GFP negative cells
  ▪ Criteria: overlap
  ▪ Output: Whole GFP negative cells
  ▪ Overlap: 80% of primary target within secondary target
  ▪ Find any matched target
• *Whole GFP positive cells*
  ▪ Primary target set: Nuclei
  ▪ Secondary target set: GFP positive cells
  ▪ Criteria: overlap
  ▪ Output: Whole GFP positive cells
  ▪ Overlap: 80% of primary target within secondary target
  ▪ Find any matched target

* Note: this step allows to subtract all the *GFP cells* target from the *Cells* target to segment the red-only cells. The end image result of *Cells*, *GFP cells* and *GFP negative cells* should be set on the 11 ^th^, 10 ^th^ and 9 ^th^ windows of the programme respectively. *GFP negative cells* is a subtraction of *GFP cells* (window 10) from *Cells* (window 11).

Protocol 2

The following protocol was used to segment two cell types in each field of view, with two distinct fluorophores. One cell type was GFP-tagged ( *GFP cells*), the other was RFP-tagged ( *RFP cells*).

***Target sets***

• *Seed*
  • Object
  • Post processing
    ▪ Erosion (Binary)
    ▪ Sieve (Binary) - greater than
• *Nuclei*
  • Nuclear segmentation
  • Post processing
    ▪ Clump breaking – second segmentation: seed
    ▪ Fill holes
    ▪ Sieve (Binary) - less than
    ▪ Sieve (Binary) - greater than
  • Measure
    ▪ Sum of Nuclei
• *RFP cells*
  • Intensity segmentation
  • Post processing
    ▪ Erosion (Binary)
    ▪ Clump breaking – second segmentation: nuclei
    ▪ Fill holes
    ▪ Sieve (Binary) - less than
    ▪ Sieve (Binary) - greater than
• *GFP cells*
  • Intensity segmentation
  • Post processing
    ▪ Erosion (Binary)
    ▪ Clump breaking – second segmentation: nuclei
    ▪ Fill holes
    ▪ Sieve (Binary) - less than
    ▪ Sieve (Binary) - greater than
• *Whole RFP cells*
  • Measure
    ▪ Count Whole RFP cells
    ▪ Sum count Whole RFP cells
    ▪ Area covered by Whole RFP cells
    ▪ Sum area covered by Whole RFP cells
• *Whole GFP cells*
  • Measure
    ▪ Count Whole GFP cells
    ▪ Sum count Whole GFP cells
    ▪ Area covered by Whole GFP cells
    ▪ Sum area covered by Whole GFP cells

***Target linking***

• *Whole RFP cells*
  ▪ Primary target set: Nuclei
  ▪ Secondary target set: RFP cells
  ▪ Criteria: overlap
  ▪ Output: Whole RFP cells
  ▪ Overlap: 80% of primary target within secondary target
  ▪ Find any matched target
• *Whole GFP cells*
  ▪ Primary target set: Nuclei
  ▪ Secondary target set: GFP cells
  ▪ Criteria: overlap
  ▪ Output: Whole GFP positive cells
  ▪ Overlap: 80% of primary target within secondary target
  ▪ Find any matched target

## Results

### Angiogenesis assay

**Figure 3.**
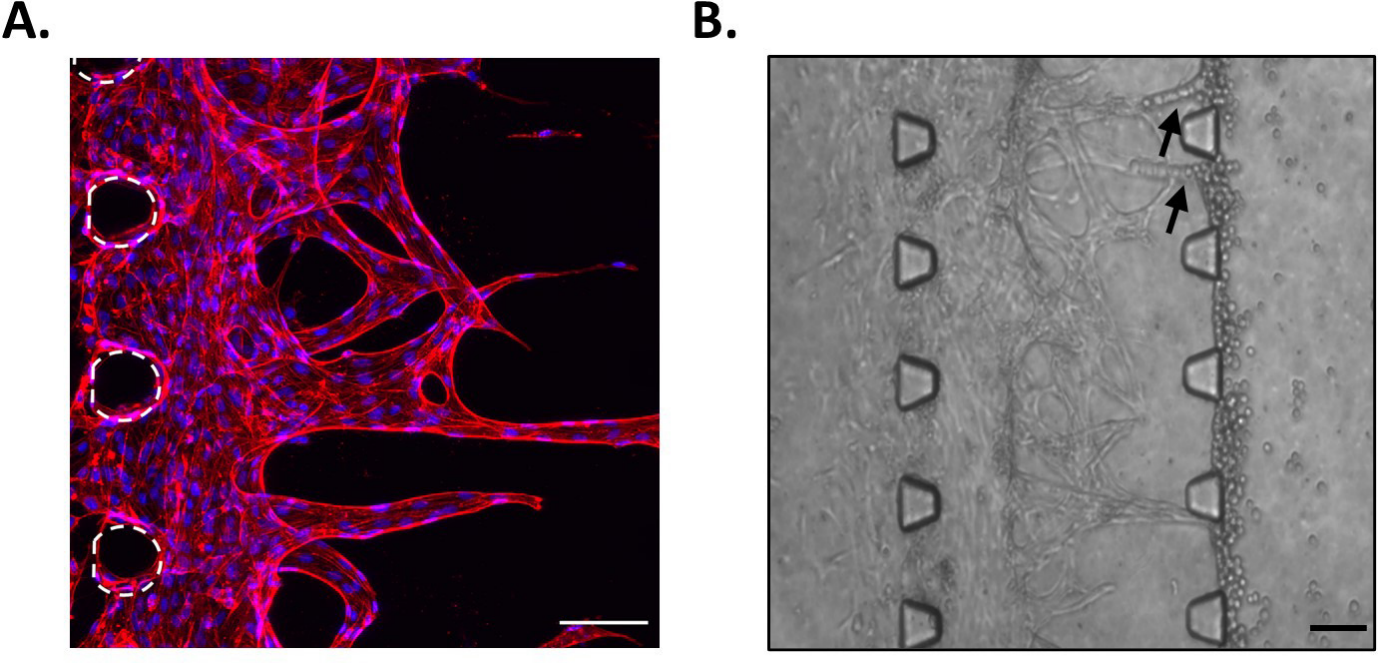
Angiogenesis assay. A. HUVECs in the middle channel of the microfluidic chip at day 10. Red, phalloidin; Blue, DAPI. Posts are highlighted in white (dashed line). Scale bar: 100 μm. B. Migration of CA1 OSCC cells into the sprouts when added in the opposite side channel. Arrows indicate cells that migrated into the sprouts. Scale bar: 100 μm.

A simple representation of the angiogenesis process was reproduced in the microfluidic chips, by adding HUVECs in the left side channel and leaving them to grow into the fibrin gel in the middle channel. Results showed that endothelial cells formed sprouts in the matrix ( Figure 3A). Furthermore, these tubular structures appeared to have a lumen, as when sprouts had grown across the whole middle channel, cells added in the opposite side channels directly migrated into these structures ( Figure 3B). Hence, by simply adding HUVECs in our device containing fibrin, we have been able to reproduce a growing vascular network, with features resembling those observed *in vivo.* This is in good agreement with comparable reports of microvascularised chips, presenting lumenated perfusable structures at this time point ( Kim *et al.*, 2013; Dibble *et al*., 2022).

### Co-culture of endothelial cells and OSCC cells to study metastasis

**Figure 4.**
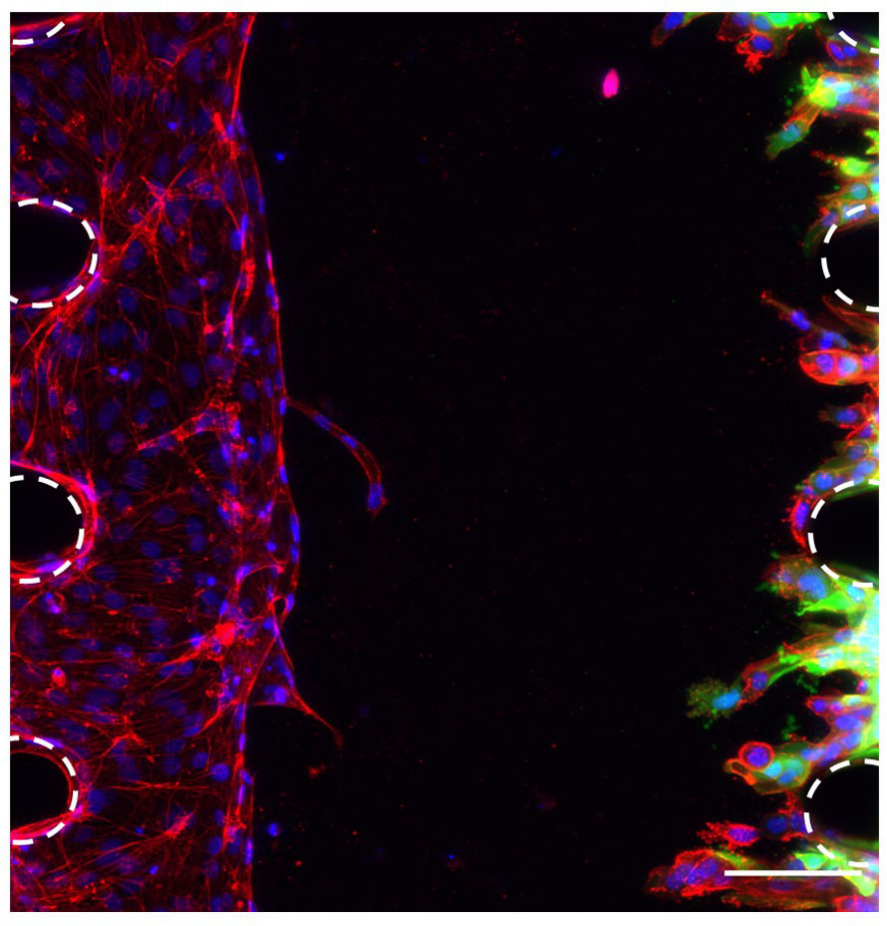
Co-culture of OSCC cells and HUVECs in the microfluidic chip. HUVECs and CA1-GFP OSCC cells in the microfluidic chip 4 days after adding the OSCC cells. Red, phalloidin; Green (GFP tag), OSCC cells; Blue, DAPI. Posts are highlighted in white (dashed line). Scale bar: 100 μm.

To study the interactions between the OSCC cells and the vasculature, co-culture of HUVECs and OSCC cells was optimised in the microfluidic chip. To allow the discrimination of these two cell types and accurate analysis of the images, GFP-tagged CA1 OSCC cells were used for this experiment ( Figure 4). These OSCC cells were added in the opposite side channel when the HUVECs had grown half way across the middle channel, as assessed daily by light microscopy (typically by day 4). Results showed that this co-culture was successful as OSCC cells invaded towards the endothelial cells in the fibrin gel ( Figure 4). This successful co-culture will enable the development of experiments to follow and assess the interactions between OSCC cells and the vascular network to better understand the metastasis of OSCC.

### Optimisation of the staining

**Figure 5.**
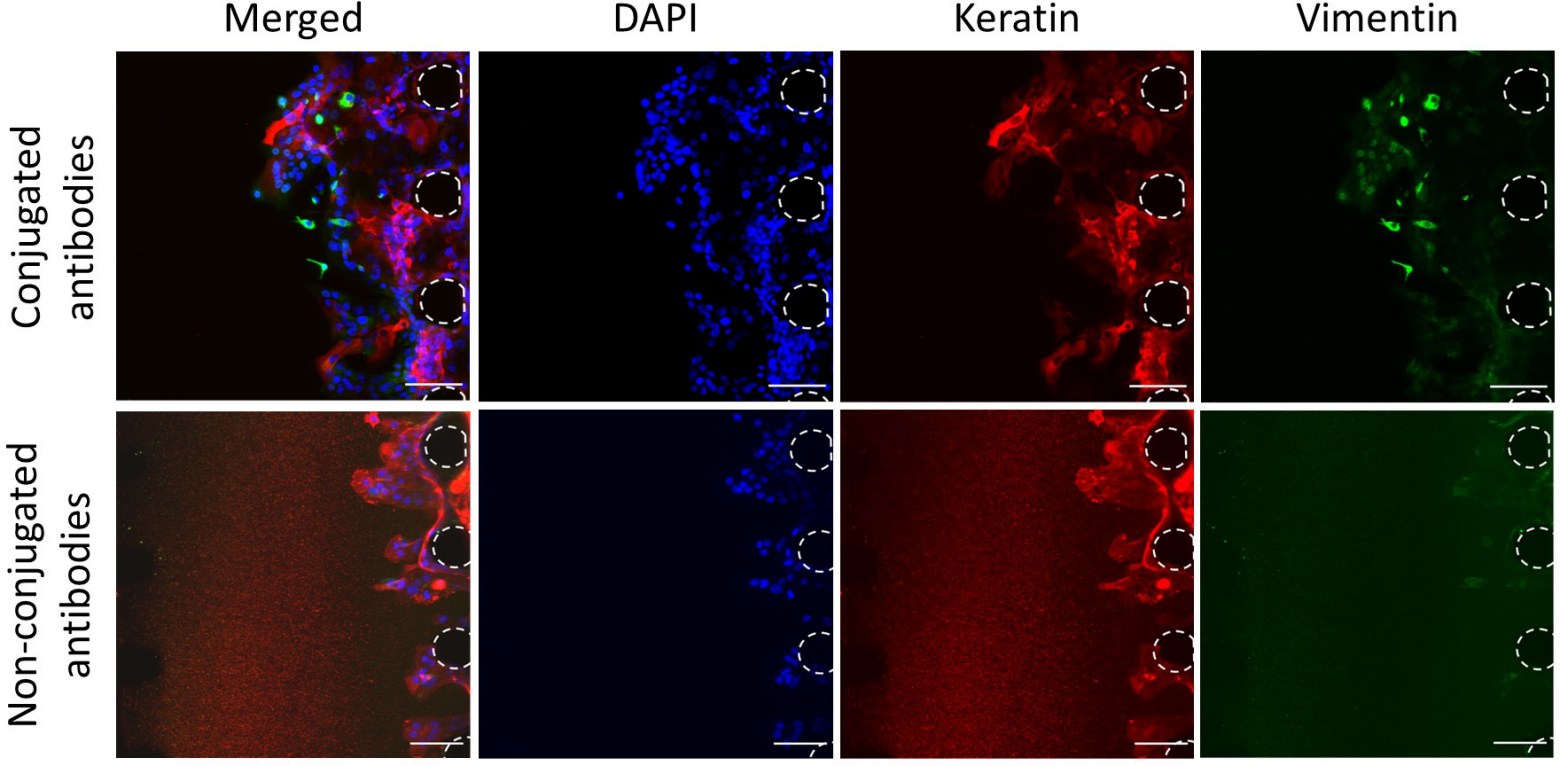
Optimisation of the staining. Staining of CA1 OSCC cells with conjugated and non-conjugated antibodies. OSCC cells without vasculature. Red, Keratin; Green, Vimentin; Blue, DAPI. Posts are highlighted in white (dashed line). Scale bars: 100 μm.

To better understand the interactions between the cancer cells and the vasculature, the expression of specific markers must be assessed. To assess the expression of markers via imaging, such as the lineage markers vimentin and keratin (marking mesenchymal and epithelial lineages respectively), antibodies are required. Non-conjugated antibodies, with primary and secondary antibodies added separately, have been widely used and optimised for imaging in biomedical research. This method allows flexibility over the fluorophore combinations used when multiple marker expressions are being assessed simultaneously. However, when tested in our microfluidic device, high background noise was detected with non-conjugated antibodies, whereas this was not the case with conjugated antibodies ( Figure 5). This background noise may reflect some retention of unbound secondary antibodies in the 3D matrix. Hence, this data shows that it may be preferable to use conjugated antibodies in microfluidic devices, to prevent high background noise and thus better analysis of the images obtained.

### Image analysis

**Figure 6.**
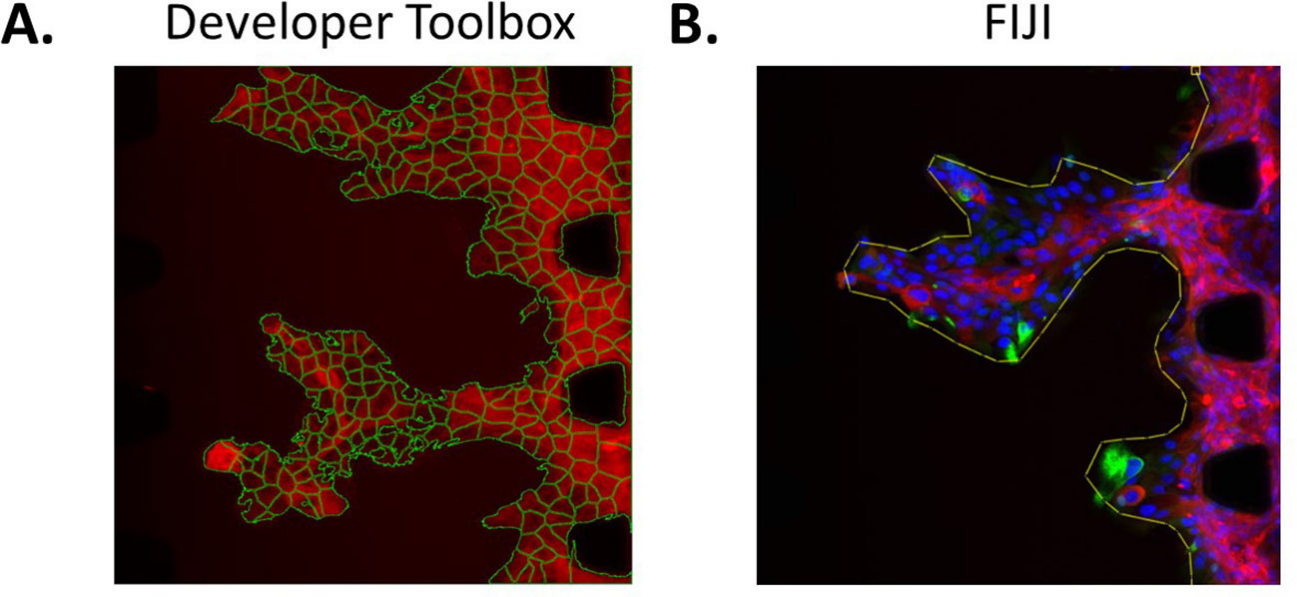
Image analysis method examples. A. Segmentation of each individual CA1-GFP cell using Developer Toolbox. B. Drawing of a region of interest in FIJI (line around the cell mass). CA1 cells stained for Vimentin (green), pan-keratin (red) and DAPI (blue).

Image analysis of the microfluidic chip experiments can be performed using several methods. For instance, for an automated approach, Developer Toolbox (GE Healthcare, version 1.10) can be employed. This programme can segment cells based on their fluorophore intensity and includes a multitude of tools enabling accurate counting of cells, assessment of the area they cover, their X position in the field of view, their shape, etc. ( Figure 6A). This automated tool can successfully segment different cell types within a field of view, as long as the cells can be differentiated with a fluorophore. For instance, in Figure 4, cancer cells are GFP-tagged and all the cells are stained with phalloidin (red). Hence, the red-only cells are endothelial cells and the red and green cells are cancer cells. Other tools can also be used for image analysis. For instance, FIJI version 2.9.0 enables drawing of regions of interest manually on each field of view, such as lines surrounding the front of the cell mass, allowing assessment of the complexity of the shape of these masses invading into the matrix ( Figure 6B).

## Discussion

The number of publications in PubMed featuring the word *organ-on-a-chip* has increased by 80-fold since the start of the century, demonstrating the high interest in this new technology for biomedical research. Indeed, microfluidic devices bring the complexity of *in vitro* models to a new level, by integrating multiple human cell types and enabling study of their interactions in a 3D environment. Furthermore, compared to animal models, microfluidic devices are characterised by their high reproducibility and controllability over the experiments ( Ma *et al.*, 2021), while allowing cellular events to be followed in real-time.

Here, a simple model of human OSCC metastasis was developed using horizontal compartmentalised microfluidic devices, with only three elements added to the device: HUVECs, OSCC cells and a fibrin gel. HUVECs were directly purchased from a manufacturer, therefore enhancing the reproducibility of this model; whereas OSCC cells were from patient-derived lines, previously characterised ( Biddle *et al.*, 2011, 2016). However, reproducibility of this system was reduced when different fibrinogen batches were used, as the animal origin of this product leads to high batch-to batch variability ( Ahadian *et al.*, 2018). Hence, for each new fibrinogen batch used, a range of fibrinogen concentrations should be tested to ensure both HUVECs and OSCC cells exhibit appropriate growth in the device. However, fibrinogen batches do not need to be replaced regularly due to the scale of these devices which allows only small volumes to be used for each experiment. The development of synthetic matrices may also counter this variability, and enables the layering of human matrix components onto the synthetic matrix to generate a fully humanised tumour environment ( Ashworth *et al*., 2020). This would further contribute to the 3Rs through reduced experimental variability and reduced reliance on animal derived components.

## Conclusions

To conclude, a new *in vitro* model to study the role of the vascular system in the metastasis of OSCC was developed and optimised, aiming to diminish the use of animal models in this field and eventually replace them. This type of device has been used for other cancer types, including breast cancer, glioblastoma, colorectal cancer, etc. ( Lee *et al.*, 2014; Jeon *et al.*, 2015; Sobrino *et al.*, 2016), demonstrating its suitability for metastasis studies regardless of the cancer cell origin. In a linked research article, currently pre-printed ( Scemama *et al.*, 2024), we have used this device to elucidate the effect of the vasculature on the invasive behaviour of OSCC cancer stem cells. The model developed here has the potential for up-scaling and adding further complexity, such as additional channels in which additional cell types and/or ECM components could be integrated, to further increase its physiological relevance ( Lee *et al.*, 2014, 2018).

## Data Availability

No data are associated with this article.
