# Supplementary material for: Development of an in vitro microfluidic model to study the role of microenvironmental cells in oral cancer metastasis
Source: F1000Res. 2024 Jan 15;12:439. Originally published 2023 Apr 25. [Version 2] doi: 10.12688/f1000research.131810.2 (PMC10904955; doi:10.12688/f1000research.131810.2)
Supplement: Peer review history

# Reviewer report: Reviewer response for version 2

**DOI:** 10.5256/f1000research.161255.r237868

**Published:** 2024-02-21

**Authors:** Christopher Jon Hanley

**Affiliations:** 1 University of Southampton, Southampton, England, UK

I have now reviewed the revised version of the manuscript and author response to my comments. With the additional detail provided and reference to the preprint demonstrating specific applications for this method I am happy to now update my status on this article to approved.

Is the rationale for developing the new method (or application) clearly explained?

Yes

Is the description of the method technically sound?

Partly

Are the conclusions about the method and its performance adequately supported by the findings presented in the article?

Partly

If any results are presented, are all the source data underlying the results available to ensure full reproducibility?

No source data required

Are sufficient details provided to allow replication of the method development and its use by others?

Partly

Reviewer Expertise:

NA

I confirm that I have read this submission and believe that I have an appropriate level of expertise to confirm that it is of an acceptable scientific standard.

# Reviewer report: Reviewer response for version 2

**DOI:** 10.5256/f1000research.161255.r237869

**Published:** 2024-02-21

**Authors:** Vivek Thacker

**Affiliations:** 1 Department of Infectious Diseases, Universitat Heidelberg (Ringgold ID: 9144), Heidelberg, Baden-Württemberg, Germany

The authors have addressed my concerns. I would only note that the description for Figure 3B is a little confusing - it is not clear to me exactly what the 'opposite channel' refers to. Are the cancer cells migrating from the left to the right channel? A minor clarification would be helpful.

Is the rationale for developing the new method (or application) clearly explained?

Yes

Is the description of the method technically sound?

Partly

Are the conclusions about the method and its performance adequately supported by the findings presented in the article?

No

If any results are presented, are all the source data underlying the results available to ensure full reproducibility?

Partly

Are sufficient details provided to allow replication of the method development and its use by others?

Partly

Reviewer Expertise:

Organ-on-chip, infectious diseases, live-cell imaging

I confirm that I have read this submission and believe that I have an appropriate level of expertise to confirm that it is of an acceptable scientific standard.

# Reviewer report: Reviewer response for version 1

**DOI:** 10.5256/f1000research.144690.r188653

**Published:** 2023-08-03

**Authors:** Christopher Jon Hanley

**Affiliations:** 1 University of Southampton, Southampton, England, UK

This article by Alice Scemama et al. outlines an in vitro approach to modelling metastatic spread in human OSCC, using microfluidic devices that enable high-quality real time imaging. The method describes the fabrication of PDMS moulds with three separate microfluidic channels that can be used for the addition of different cells, matrices or media to the culture. Protocols are also described for imaging-based analysis of these cultures using endogenous fluorophores and/or antibody staining followed by digital analysis involving cell segmentation and quantification of fluorescence intensity.

 The method provides useful insight for device fabrication and use for culturing OSCC cells with endothelial cells. As stated by the authors, this device and culture system replicates others that have been used for studying different cancer types previously, so these insights are not entirely novel. It also remains questionable to what degree the stated research highlights are fully achieved by this article.

 Conceptual reservations to approval of this article:

- The authors propose that this microfluidic system could be used for investigating the process of OSCC metastasis via the vasculature, suggesting that this could be used instead of animal models. To adequately demonstrate this, further evidence of benchmarking compared to alternative *in vitro* systems and *in vivo* models is required. For example, additional experiments or data that would be useful include:
  - Demonstrating that cell lines with varying metastatic potential *in vivo* exhibit different phenotypes in this model.
  - Demonstrating quantitative metrics can be derived from this model system and the imaging-based analyses proposed to assess these phenotypic differences.
  - Comparing the results generated using this system to a Transwell system or other existing systems (e.g. Ibidi chemotaxis slides).
- The authors highlight real time imaging as a major advantage of this system over existing methods for studying OSCC metastasis. However, the data presented exposes some limitations to these approaches and their utility for progressing the investigation of molecular mechanisms underlying these processes. For example,
  - The use of endogenous fluorophores and phalloidin for classifying different cell types is confounded by non-ubiquitous reporter expression leading to potential for mis-classification of cells, which appears to be the case in the micrograph shown in Figure 4.
  - The interpretation of intercellular interactions across cellular compartments is reliant on strict lineage specific restriction of marker expression. The example provided in figure 5 demonstrates the use of keratin and vimentin “to better understand the interactions between the cancer cells and the vasculature”. However, the use of vimentin here is likely to be suboptimal, given this gene is expressed in cancer cells undergoing EMT. The authors should elaborate further on how experiments should be designed to enable accurate examination of tumour vasculature interactions and ensure that the interpretations are validated with appropriate controls.
  - In addition (and as described above) it is not clear how these imaging-based analyses should be used to quantify steps in the metastatic process. Further evaluation and demonstration  of how these approaches can be used to measure phenotypic changes under different experimental conditions should be provided to confirm this system be used in a similar manner to existing assays (e.g. Transwell systems) generating reproducible quantitative data.

Technical recommendations/comments:

 In the Angiogenesis assay section:

 “cells added in the opposite side channels directly migrated into these structures” – clarity should be provided regarding what cells were added to opposite side in this example (Figure 3).

 In the Co-culture of endothelial cells and OSCC cells to study metastasis section:

 “Results showed that this co-culture was successful as OSCC cells invaded towards the endothelial cells in the fibrin gel” - can the authors explain why angiogenic sprouting was not observed in the cancer cell co-culture? Also, for this statement to be valid it should be shown that invasion into the fibrin gel does not occur in monoculture; in the subsequent figures where OSCC cells are shown alone there is evidence of invasion suggesting this process may be independent of the presence of endothelial cells.

 In the Optimisation of the staining section:

 “To better understand the interactions between the cancer cells and the vasculature, the expression of specific markers must be assessed” – as described above the markers chosen (keratin and vimentin) are not appropriate for this purpose, use of CD31 or an alternative endothelial cell specific marker should be used. Further clarity should also be provided in the legend for figure 5. Is this example showing OSCC cells grown in monoculture or co-culture?

 “This background noise may reflect some retention of unbound secondary antibodies in the 3D matrix.” -  can the authors provide rationale for why excess secondary antibody but not primary antibody would be retained within the 3D matrix? Could indirect antibody staining be feasible with further optimisation of the staining protocol? If conjugated antibodies must be used instead of indirect antibody staining the feasibility for analysing different proteins in this system is likely to be significantly reduced, due to limited commercial availability of conjugated antibodies and lack of signal amplification.

 In the Image analysis section:

 The description of cell segmentation and analysis methodology is not adequately described for readers unfamiliar with the developer toolbox software. Clear descriptions should be provided for the algorithms used for cell segmentation. Additionally, it is unclear whether the term cell segmentation is used correctly throughout the article. This process refers to demarcation of cellular boundaries but seems to be used to describe both cell segmentation and cell-type/group classification, which are two distinct processes.

Is the rationale for developing the new method (or application) clearly explained?

Yes

Is the description of the method technically sound?

Partly

Are the conclusions about the method and its performance adequately supported by the findings presented in the article?

Partly

If any results are presented, are all the source data underlying the results available to ensure full reproducibility?

No source data required

Are sufficient details provided to allow replication of the method development and its use by others?

Partly

Reviewer Expertise:

Tumour microenvironment research using 3D cultures, digital pathology and bioinformatics

I confirm that I have read this submission and believe that I have an appropriate level of expertise to confirm that it is of an acceptable scientific standard, however I have significant reservations, as outlined above.

# Response

**Published:** 2024-01-10

**Authors:** Adrian Biddle

*This article by Alice Scemama et al. outlines an in vitro approach to modelling metastatic spread in human OSCC, using microfluidic devices that enable high-quality real time imaging. The method describes the fabrication of PDMS moulds with three separate microfluidic channels that can be used for the addition of different cells, matrices or media to the culture. Protocols are also described for imaging-based analysis of these cultures using endogenous fluorophores and/or antibody staining followed by digital analysis involving cell segmentation and quantification of fluorescence intensity.*

*The method provides useful insight for device fabrication and use for culturing OSCC cells with endothelial cells. As stated by the authors, this device and culture system replicates others that have been used for studying different cancer types previously, so these insights are not entirely novel. It also remains questionable to what degree the stated research highlights are fully achieved by this article.*

 We thank the reviewer for their encouraging words and thorough review. As mentioned for reviewer 1, this is a methods paper to accompany a research article that is now pre-printed (Scemama et al., 2024). Whilst conducting patient validation of the data from the microfluidic device for the main research article, we wanted to publish the method without delay as a resource for the research community. We have now updated this methods paper to link it to the pre-printed research article. Where comments are addressed in the main research article, we will note that below.

 The particular focus of our own work is on using microfluidic devices to study the evolution of tumour cellular heterogeneity and plasticity within a complex tumour microenvironment, and the analysis methods we have developed and presented here are designed to enable the application of microfluidic devices to this area of study.

*Conceptual reservations to approval of this article:*

- *The authors propose that this microfluidic system could be used for investigating the process of OSCC metastasis via the vasculature, suggesting that this could be used instead of animal models. To adequately demonstrate this, further evidence of benchmarking compared to alternative in vitro systems and in vivo models is required. For example, additional experiments or data that would be useful include:*
  - *Demonstrating that cell lines with varying metastatic potential in vivo exhibit different phenotypes in this model.*
  - *Demonstrating quantitative metrics can be derived from this model system and the imaging-based analyses proposed to assess these phenotypic differences.*
  - *Comparing the results generated using this system to a Transwell system or other existing systems (e.g. Ibidi chemotaxis slides).*

An important advantage of this system is the ability to generate quantitative single-cell metrics, due to the superior imaging over existing *in vitro* and *in vivo* methods. Methods for the generation of such metrics are presented in this methods paper, and we have presented both the outputs of these methods and patient validation data in the main research article (Scemama et al., 2024). Our focus is on benchmarking against human clinical and pathological data, as *in vivo *animal data is often an inaccurate representation of the clinical truth.

  

- *The authors highlight real time imaging as a major advantage of this system over existing methods for studying OSCC metastasis. However, the data presented exposes some limitations to these approaches and their utility for progressing the investigation of molecular mechanisms underlying these processes. For example,*
  - *The use of endogenous fluorophores and phalloidin for classifying different cell types is confounded by non-ubiquitous reporter expression leading to potential for mis-classification of cells, which appears to be the case in the micrograph shown in Figure 4.*

Reviewer 1 had the same concern, and we have responded in detail there. Briefly, this concern is due to the inability to display the entire dynamic expression range in an image. The expression is in fact ubiquitous.

  

-
  - *The interpretation of intercellular interactions across cellular compartments is reliant on strict lineage specific restriction of marker expression. The example provided in figure 5 demonstrates the use of keratin and vimentin “to better understand the interactions between the cancer cells and the vasculature”. However, the use of vimentin here is likely to be suboptimal, given this gene is expressed in cancer cells undergoing EMT. The authors should elaborate further on how experiments should be designed to enable accurate examination of tumour vasculature interactions and ensure that the interpretations are validated with appropriate controls.*

The reference to keratin and vimentin expression here is in relation to the cancer cells, as we are interested in identifying cancer cells undergoing EMT. The vascular cells do indeed also express vimentin, but this can be separated out by including co-localisation with the GFP lineage reporter in the quantitative analysis. This is elaborated further in the associated research article (Scemama et al., 2024).

  

-
  - *In addition (and as described above) it is not clear how these imaging-based analyses should be used to quantify steps in the metastatic process. Further evaluation and demonstration of how these approaches can be used to measure phenotypic changes under different experimental conditions should be provided to confirm this system be used in a similar manner to existing assays (e.g. Transwell systems) generating reproducible quantitative data.*

The assessment of changes under different experimental conditions, using the quantitative methods described in this methods paper, is a key feature of the associated research article (Scemama et al., 2024).

*Technical recommendations/comments:*

*In the Angiogenesis assay section:*

*“cells added in the opposite side channels directly migrated into these structures” – clarity should be provided regarding what cells were added to opposite side in this example (Figure 3).*

 The cells in the opposite channel were CA1, and we have now clarified this.

*In the Co-culture of endothelial cells and OSCC cells to study metastasis section:*

*“Results showed that this co-culture was successful as OSCC cells invaded towards the endothelial cells in the fibrin gel” - can the authors explain why angiogenic sprouting was not observed in the cancer cell co-culture? Also, for this statement to be valid it should be shown that invasion into the fibrin gel does not occur in monoculture; in the subsequent figures where OSCC cells are shown alone there is evidence of invasion suggesting this process may be independent of the presence of endothelial cells.*

 Indeed, the invasion of CA1 cells into the device occurs in both the presence and absence of HUVECs. Our point here is that we have established culture conditions that allow the successful co-culture of both cell types within the device such that their interactions can be investigated. Investigation of the effects of this co-culture on both the cancer and endothelial cells is a major focus of the associated research article (Scemama et al., 2024).

*In the Optimisation of the staining section:*

*“To better understand the interactions between the cancer cells and the vasculature, the expression of specific markers must be assessed” – as described above the markers chosen (keratin and vimentin) are not appropriate for this purpose, use of CD31 or an alternative endothelial cell specific marker should be used. Further clarity should also be provided in the legend for figure 5. Is this example showing OSCC cells grown in monoculture or co-culture?*

 This example of staining is on cells growing in monoculture – this has been added. As we’ve outlined in a previous answer, vimentin and keratin are both used as markers in the cancer cells. CD31 can successfully mark the endothelial cells, as can an RFP marker (Scemama et al., 2024).

*“This background noise may reflect some retention of unbound secondary antibodies in the 3D matrix.” -  can the authors provide rationale for why excess secondary antibody but not primary antibody would be retained within the 3D matrix? Could indirect antibody staining be feasible with further optimisation of the staining protocol? If conjugated antibodies must be used instead of indirect antibody staining the feasibility for analysing different proteins in this system is likely to be significantly reduced, due to limited commercial availability of conjugated antibodies and lack of signal amplification.*

 We tried extensive optimisation of indirect antibody staining before proceeding with direct antibody staining so, whilst we cannot know whether it would be feasible with the correct optimisation, it certainly seems a difficult approach within this system. In contrast, direct antibody staining worked very well. We suggest that, being a 3D system, there is more opportunity for antibody to diffuse away from the intended target than in 2D staining. Certainly, extended time between staining and imaging increases this problem. It may be that the binding of primary antibodies to their target is stronger than the binding of secondary antibodies, with less diffusion away, and of course there is less fluorescence signal overall in the system.

 In the past, we have relied on indirect staining due to the signal amplification effect and limited commercial availability of conjugated antibodies. However, these advantages of indirect staining no longer hold. Improved imaging systems and techniques have rendered the signal amplification effect of secondary antibodies unnecessary for our applications, and commercial providers now have greatly improved catalogues of conjugated antibodies (including recombinantly produced antibodies) such that the majority of targets of interest have antibodies available conjugated to a range of fluorophores.

*In the Image analysis section:*

*The description of cell segmentation and analysis methodology is not adequately described for readers unfamiliar with the developer toolbox software. Clear descriptions should be provided for the algorithms used for cell segmentation. Additionally, it is unclear whether the term cell segmentation is used correctly throughout the article. This process refers to demarcation of cellular boundaries but seems to be used to describe both cell segmentation and cell-type/group classification, which are two distinct processes.*

 We have updated this section to improve the description and clearly define the term ‘segmentation’.

 References:

 SCEMAMA, A., LUNETTO, S., TAILOR, A., CIO, S. D., AMBLER, L., COETZEE, A., COTTOM, H., KHURRAM, S. A., GAUTROT, J. & BIDDLE, A. 2024. Hybrid cancer stem cells utilise vascular tracks for collective streaming invasion in a metastasis-on-a-chip device. *bioRxiv* **,** 2024.01.02.573897.

# Reviewer report: Reviewer response for version 1

**DOI:** 10.5256/f1000research.144690.r171696

**Published:** 2023-06-15

**Authors:** Vivek Thacker

**Affiliations:** 1 Department of Infectious Diseases, Universitat Heidelberg (Ringgold ID: 9144), Heidelberg, Baden-Württemberg, Germany

In this manuscript, Scemama and colleagues describe the design, fabrication, and testing of an organ-on-chip device to visualize the metastasis of patient-derived oral squamous carcinoma cells into vascular networks. The device consists of vascular cells seeded in one channel that form an extended network into the adjacent (central) fibrin-gel containing channel and OSCC cells seeded in the other side channel that interact with the vascular cells in the central channel. The use of such a side-by-side geometry overcomes many of the limitations of layered microfluidic devices. OSCC cells are identified by GFP transduction. Overall, the rationale for the need for such devices is clearly described, and the fabrication processes of the organ-chip device has been described with sufficient detail to enable to reader to replicate these experiments. However, there are shortcomings in relation to the characterization of the devices from a cellular perspective. The availability of patient-derived OSCC cells is a huge asset, but apart from the CA1 line, none of the others described in Table 1 are used in this study. There are several similar designs reported for the culture of vascular networks in co-culture with several other cell types including tumour cells, and the authors should place their device in the context of this work. In addition, the authors do not show evidence for a close co-culture in the central channel between the endothelial and OSCC cells, which is needed if this device is to be used to study metastasis as claimed.

**Major comments:**

**Characterization of GFP-labelled OSCC cells** – the data in Figure 4 suggests that several of the GFP labelled cells have low or no GFP expression whereas others have high GFP expression. Did the authors choose one particular clone for expansion or are the cells a mixed population? Protocols for characterization of these cells are important and should be included so as to enable others to repeat these experiments. Furthermore, the lack of GFP expression in a sub-population of OSCC cells is problematic given that this marker is used to classify the OSCC cells in the chip. Are these cells losing GFP expression over the period of growth in the organ-on-chip? The authors could show data with other epithelial markers like EpCAM or E-cadherin to characterize these cells further.

**Co-culture on-chip – **the manuscript claims in Figures 2 (v) and (vi) that a co-culture of a vascular network and the OSCC cells is possible, but this is not supported by the images in Figures 4, 5, and 6. The timepoint at which the chips are imaged in Figures 4, 5, and 6 are not clear – it is likely that these are early timepoints and there has been insufficient time to generate the co-culture. Evidence from later stages needs to be provided to back up this claim.

**Vascular network on-chip** – the authors claim on page 14 and in relation to Figure 3 that their protocol generates a perfusable vascular network, but there is no functional evidence provided. Please nuance this claim or provide evidence to support it. Furthermore, the authors do not adequately cite other publications in this field, notably from the lab of Prof Roger Kamm who has created several models that have extensive perfusable vascularization, including with the addition of tumor cells. The authors should place their model in the context of this literature.

**Table 1 and OSCC cells** – the need for table 1 is unclear since none of the other patient-derived cell lines are used in this study and the protocols that the authors propose requires the use of the GFP-labelling to identify OSCC cells. Inclusion of the patient-derived cells is a strength, and the authors could significantly strengthen the manuscript by comparing the behaviours of these four cell lines in the same device for parameters such as invasion or growth or spread via metastasis.

**Image analysis pipeline** – it is unclear how the images generated in Figure 6 are obtained, and how this is useful for the purpose of analysis of metastasis. It is unclear how the cell boundaries in 6A are determined given the low and homogenous GFP expression. This image doesn’t quite correlate with the image in 6B, is this a different field of view? In 6B there are very few GFP+ cells, so is this an area from the vascular side of the device? In any case, it does not appear that a vascular network that interacts with many OSCC cells (as depicted in Figure 2 (vi)) has been generated.

**Minor comments:**

**Page 4, limitations of in vivo models **- several limitations are described at the top of page 4, it would be useful to have references to these.

**Page 4, limitations of transwells – **these models are described as unsuitable for real-time imaging. The authors should clarify exactly why (transparency of membrane, working distance etc.) to help the reader assess these limitations.****

**Page 5, UV light – **typo, should be mW and not Mw.

**Figure 1  - **the schematics are nicely presented, showing an XZ or YZ cross-section would also help the reader appreciate the low working distance from the coverslip afforded by the authors’ design.

**Page 7 – **the details of the vector used for GFP expression are incompletely described. Please describe these and provide references for the vector. Typo – ‘polybrene’ and not ‘prolybrene’.

**Figures 3-6** – please avoid the use of primary colours to enable colour-blind readers to appreciate the images.

Is the rationale for developing the new method (or application) clearly explained?

Yes

Is the description of the method technically sound?

Partly

Are the conclusions about the method and its performance adequately supported by the findings presented in the article?

No

If any results are presented, are all the source data underlying the results available to ensure full reproducibility?

Partly

Are sufficient details provided to allow replication of the method development and its use by others?

Partly

Reviewer Expertise:

Organ-on-chip, infectious diseases, live-cell imaging

I confirm that I have read this submission and believe that I have an appropriate level of expertise to confirm that it is of an acceptable scientific standard, however I have significant reservations, as outlined above.

# Response

**Published:** 2024-01-10

**Authors:** Adrian Biddle

*In this manuscript, Scemama and colleagues describe the design, fabrication, and testing of an organ-on-chip device to visualize the metastasis of patient-derived oral squamous carcinoma cells into vascular networks. The device consists of vascular cells seeded in one channel that form an extended network into the adjacent (central) fibrin-gel containing channel and OSCC cells seeded in the other side channel that interact with the vascular cells in the central channel. The use of such a side-by-side geometry overcomes many of the limitations of layered microfluidic devices. OSCC cells are identified by GFP transduction. Overall, the rationale for the need for such devices is clearly described, and the fabrication processes of the organ-chip device has been described with sufficient detail to enable to reader to replicate these experiments. However, there are shortcomings in relation to the characterization of the devices from a cellular perspective. The availability of patient-derived OSCC cells is a huge asset, but apart from the CA1 line, none of the others described in Table 1 are used in this study. There are several similar designs reported for the culture of vascular networks in co-culture with several other cell types including tumour cells, and the authors should place their device in the context of this work. In addition, the authors do not show evidence for a close co-culture in the central channel between the endothelial and OSCC cells, which is needed if this device is to be used to study metastasis as claimed.*

 We thank the reviewer for their enthusiasm for the method presented in our report, and for their thorough review. We will answer the specific comments where they appear below. One thing to note is that this is a methods paper to accompany a research article that is now available as a pre-print (Scemama et al., 2024). Whilst conducting patient validation of the data from the microfluidic device for the main research article, we wanted to publish the method without delay as a resource for the research community. We have now updated this methods paper to link it to the pre-printed research article. Where comments are addressed in the main research article, we will note that below.

***Major comments:***

***Characterization of GFP-labelled OSCC cells** – the data in Figure 4 suggests that several of the GFP labelled cells have low or no GFP expression whereas others have high GFP expression. Did the authors choose one particular clone for expansion or are the cells a mixed population? Protocols for characterization of these cells are important and should be included so as to enable others to repeat these experiments. Furthermore, the lack of GFP expression in a sub-population of OSCC cells is problematic given that this marker is used to classify the OSCC cells in the chip. Are these cells losing GFP expression over the period of growth in the organ-on-chip? The authors could show data with other epithelial markers like EpCAM or E-cadherin to characterize these cells further.*

 The apparent lack of GFP expression in some OSCC cells in Fig. 4 is due to the fact that the level of GFP expression is over enough of a range that the levels in the lower expressing cells cannot be visualised without making the higher expressing cells too bright. Quantitative analysis of GFP expression compared to a negative control (e.g. HUVEC) demonstrates that it is positive in all of the OSCC cells. It is also positive in all OSCC cells when tested by flow cytometry.

 The protocol for production of GFP+ CA1 cells, and their characterisation by flow cytometry, is detailed in the cited publication (Gemenetzidis et al., 2015). We do not use clonal expansion, as the cancer cell line is a heterogeneous population and therefore single cell expansion results in a non-representative population.

 EpCAM and E-cadherin expression are investigated in the associated research article (Scemama et al., 2024), and also exhibit a range of expression levels in the OSCC cells.

***Co-culture on-chip – **the manuscript claims in Figures 2 (v) and (vi) that a co-culture of a vascular network and the OSCC cells is possible, but this is not supported by the images in Figures 4, 5, and 6. The timepoint at which the chips are imaged in Figures 4, 5, and 6 are not clear – it is likely that these are early timepoints and there has been insufficient time to generate the co-culture. Evidence from later stages needs to be provided to back up this claim.*

 The timepoints are noted in the figure legends. The evolution of the co-culture after addition of OSCC cells is the specific focus of the associated research article, and is described in detail there (Scemama et al., 2024). The purpose of this methods article is to describe methods for production of the co-culture devices and for quantitative analysis of the resulting cultures. Briefly, the separation of OSCC cells and HUVECs is due to their response to one another. Allowing more complete vascularisation before adding OSCC cells results in OSCC cells flowing through the vasculature (Fig. 3), which may or may not be desirable depending on study aims.

***Vascular network on-chip** – the authors claim on page 14 and in relation to Figure 3 that their protocol generates a perfusable vascular network, but there is no functional evidence provided. Please nuance this claim or provide evidence to support it. Furthermore, the authors do not adequately cite other publications in this field, notably from the lab of Prof Roger Kamm who has created several models that have extensive perfusable vascularization, including with the addition of tumor cells. The authors should place their model in the context of this literature.*

 The functional evidence for the production of a lumenised vasculature is the image in Fig. 3B showing cancer cells flowing directly into the vascular lumen. This happens when we allow the vasculature to progress across the whole width of the middle channel and connect to the far side channel, before adding the cancer cells. Usually, we add the cancer cells before the vasculature reaches this level of development, as shown in the other images presented.

 We agree that our work builds on important previous work on the development of these microfluidic devices for investigation of cancer interaction with the vasculature. Prof Roger Kamm pioneered these devices, and we have cited his work (Jeon et al., 2015) alongside other key previous studies. This is in the conclusion section in page 17, and in the first results section relating to previous studies demonstrating production of perfusable vascular structures. The particular focus of our own work is on using these devices to study the evolution of tumour cellular heterogeneity and plasticity within a complex tumour microenvironment, and the analysis methods we have developed and presented here are designed to enable the application of microfluidic devices to this area of study.

***Table 1 and OSCC cells** – the need for table 1 is unclear since none of the other patient-derived cell lines are used in this study and the protocols that the authors propose requires the use of the GFP-labelling to identify OSCC cells. Inclusion of the patient-derived cells is a strength, and the authors could significantly strengthen the manuscript by comparing the behaviours of these four cell lines in the same device for parameters such as invasion or growth or spread via metastasis.*

 The inclusion of cell lines not used in this methods paper was a mistake, and we have now removed them. These are the cell lines used in the associated research article (Scemama et al., 2024), and we agree that the inclusion of multiple patient-derived cell lines is an important consideration for research studies using these devices.

***Image analysis pipeline** – it is unclear how the images generated in Figure 6 are obtained, and how this is useful for the purpose of analysis of metastasis. It is unclear how the cell boundaries in 6A are determined given the low and homogenous GFP expression. This image doesn’t quite correlate with the image in 6B, is this a different field of view? In 6B there are very few GFP+ cells, so is this an area from the vascular side of the device? In any case, it does not appear that a vascular network that interacts with many OSCC cells (as depicted in Figure 2 (vi)) has been generated.*

 These images are of control devices with no vasculature. The purpose of this figure was to show the analysis methods used, we therefore used images of OSCC monoculture to make it easier to follow.

 In 6a, the cells are CA1-GFP. This is to show that with fluorescent cells, Developer can easily be used to segment the cells using the nucleus (DAPI staining).

 In 6b, the cells are stained with Vimentin/Keratin with Vimentin in green and Keratin in red. The cells used here were normal CA1 (non-GFP CA1) in order to be able to do the double antibody stain.

 The legend has been updated to make the above details clear.

 These two images are indeed different but this is because the goal was to show two different methods

 1) With fluorescent CA1, it is a lot easier to do an automatic segmentation (as shown in 6a).

 2) With non-fluorescent CA1, stained for markers, a manual analysis is preferred (6b). Here we are showing how to calculate the length of the cell front by drawing a line manually.

***Minor comments:***

***Page 4, limitations of in vivo models **- several limitations are described at the top of page 4, it would be useful to have references to these.*

 The reference has been moved to make clear it covers all of these limitations. It is a review that covers these in more detail.

***Page 4, limitations of transwells – **these models are described as unsuitable for real-time imaging. The authors should clarify exactly why (transparency of membrane, working distance etc.) to help the reader assess these limitations.*****

 This has been updated with further explanation.

***Page 5, UV light – **typo, should be mW and not Mw.*

 Updated.

***Figure 1  - **the schematics are nicely presented, showing an XZ or YZ cross-section would also help the reader appreciate the low working distance from the coverslip afforded by the authors’ design.*

 This is a good point; we have edited the legend to add this point.

***Page 7 – **the details of the vector used for GFP expression are incompletely described. Please describe these and provide references for the vector. Typo – ‘polybrene’ and not ‘prolybrene’.*

 We have re-considered the inclusion of this methods section, as the GFP line was produced as part of a previous study and not by the authors of the current study. We have instead included a reference to the study where the CA1-GFP were produced (Gemenetzidis et al., 2015).

***Figures 3-6** – please avoid the use of primary colours to enable colour-blind readers to appreciate the images.*

 Thank you. We will incorporate this point into our future image analysis pipeline.

 References

 GEMENETZIDIS, E., GAMMON, L., BIDDLE, A., EMICH, H. & MACKENZIE, I. C. 2015. Invasive oral cancer stem cells display resistance to ionising radiation. *Oncotarget,* 6 **,** 43964-77.

 SCEMAMA, A., LUNETTO, S., TAILOR, A., CIO, S. D., AMBLER, L., COETZEE, A., COTTOM, H., KHURRAM, S. A., GAUTROT, J. & BIDDLE, A. 2024. Hybrid cancer stem cells utilise vascular tracks for collective streaming invasion in a metastasis-on-a-chip device. *bioRxiv* **,** 2024.01.02.573897.
